# Decrease in Vitamin D Status in the Greenlandic Adult Population from 1987–2010

**DOI:** 10.1371/journal.pone.0112949

**Published:** 2014-12-02

**Authors:** Nina O. Nielsen, Marit E. Jørgensen, Henrik Friis, Mads Melbye, Bolette Soborg, Charlotte Jeppesen, Marika Lundqvist, Arieh Cohen, David M. Hougaard, Peter Bjerregaard

**Affiliations:** 1 National Institute of Public Health, University of Southern Denmark, Copenhagen, Denmark; 2 Steno Diabetes Centre, Gentofte, Denmark; 3 Department of Nutrition, Exercise and Sports, University of Copenhagen, Copenhagen, Denmark; 4 Department of Epidemiology Research, Statens Serum Institut, Copenhagen, Denmark; 5 Department of Public Health, Aarhus University, Aarhus, Denmark; 6 Department of Clinical Biochemistry and Immunology, Statens Serum Institut, Copenhagen, Denmark; 7 Greenland Centre for Health Research, University of Greenland, Nuuk, Greenland; University of Tennessee, United States of America

## Abstract

**Background:**

Low vitamin D status may be pronounced in Arctic populations due to limited sun exposure and decreasing intake of traditional food.

**Objective:**

To investigate serum 25(OH)D3 as a measure of vitamin D status among adult Inuit in Greenland, predictors of low serum 25(OH)D3 concentrations and the trend from 1987 to 2005–2010.

**Design:**

A total of 2877 randomly selected Inuit (≥18 years) from the Inuit Health in Transition study were included. A sub-sample (n = 330) donated a blood sample in 1987 which allowed assessment of time trends in vitamin D status.

**Results:**

The geometric mean serum 25(OH)D3 (25[OH]D2 concentrations were negligible and not reported) in 2005–2010 was lowest among the 18–29 year old individuals (30.7 nmol/L; 95% CI: 29.7; 31.7) and increased with age. In all age-groups it decreased from 1987 to 2005–2010 (32%–58%). Low 25(OH)D3 concentrations (<50 nmol/L) were present in 77% of the 18–29 year old and decreased with age. A characteristic seasonal variation in 25(OH)D3 concentrations was observed (range 33.2–57.1 nmol/L, p<0.001), with the highest concentrations in August to October. Age (2.0% per year increase; CI: 1.7, 2.2), female gender (7.1%; CI: 2.0; 12.5), alcohol intake (0.2% per increase in drinks/week; 0.0; 0.4), and traditional diet (10.0% per 100 g/d increase; CI: 7.9; 12.1) were associated with increased serum 25(OH)D3, whereas smoking (−11.6%; CI: −16.2; −6.9), BMI (−0.6%; CI: −1.1; −0.2) and latitude (−0.7% per degree increase; CI: −1.3; −0.2) were associated with decreased concentrations.

**Conclusion:**

We identified a remarkable decrease in vitamin D status from 1987 to 2005–2010 and a presently low vitamin D status among Inuit in Greenland. A change away from a traditional diet may well explain the observed decline. The study argues for the need of increased dietary intake of vitamin D and supplementation might be considered.

## Introduction

The Inuit population in Greenland has been exposed to an extensive nutrition- and health transition during the past 50–60 years [1], [2]. The traditional diet containing fish, sea mammals, local plants and berries, has to a large extent been substituted by imported meat, sweets, chips, cakes and soft drinks. Furthermore, a physically active lifestyle characterized by fishing and hunting has changed to a more sedentary way of living [3]. This has been accompanied by an increased body weight and prevalence of type 2 diabetes mellitus [4], [5] and possibly cardiovascular diseases [6].

Worldwide, epidemiological evidence suggest an association between low vitamin D status and metabolic and cardiovascular disorders, as well as infectious and inflammatory diseases [7]–[12], indicating a potential important role of vitamin D in human health. Populations in the Arctic are subject to low vitamin D synthesis in the skin due to the extended periods of darkness, and during summer season due to high solar zenith angle [13] and continued need for outdoor clothing [14]. Furthermore, the Inuit skin pigmentation may reduce vitamin D synthesis. Formerly, vitamin D intake through the traditional diet probably partially compensated for the poor skin synthesis. The traditional dietary sources of vitamin D are mainly fatty fish and products from seal and whale [15], [16]. Imported foods such as eggs and high-fat dairy products contain only little vitamin D, unlikely to cover the biological requirements. Thus, individuals living in the Arctic and relying on a western diet appear to be at particularly high risk of low vitamin D status.

So far only three small studies have reported on vitamin D concentrations in a Greenlandic population. These have either dealt with a specific geographic area of Greenland [14], [17], [18] or described associations with a health outcome [11]. We aimed to examine the vitamin D status in a large and representative sample of the adult Inuit population from all geographical areas in Greenland recruited in the period 2005–2010. Furthermore, to evaluate the hypothesis that vitamin D status has decreased during the last few decades, we examined vitamin D concentrations in a subsample of this population, from whom a blood sample was available from 1987, and estimated the change in vitamin D status from 1987 to 2005–2010. A secondary aim was to identify predictors of vitamin D status among Inuit in Greenland.

## Subjects And Methods

### Study participants and design

Greenland is the world's largest island (2,175,600 km^2^) of which 85% is covered by an ice cap and only a narrow coastal strip is inhabited. There are 16 towns and approximately 60 settlements. Approximately 90% of the 57.000 inhabitants are Inuit, while the remaining are mainly Danes.

Study participants were indigenous Greenlanders (Inuit), based on the primary language and self-identification, selected as a stratified random sample based on population lists from the central population register as described elsewhere [19], [20]. Participants were part of the Inuit Health in Transition (IHIT) study, a general health study among adults (≥18 years) in Greenland established in 2005–2010 to allow investigations of health and diseases, lifestyle and life conditions [20]. A total of 3108 adult Inuit (9.1% of the total adult population in 2005) representing all age groups above 18 years, all geographical areas (latitudes ranging from 59,98 degrees north to 77,46 degrees north) and all community sizes, were included. From 2877 (93%), a serum 25-hydroxyvitamin D3 (25(OH)D3) measurement was obtained, and these were included in a cross sectional study. Among these, 485 individuals had participated in a population-based serological survey in 1987 carried out to screen for syphilis in western and southern districts of Greenland [21]. The syphilis screening, and treatment, was offered to all individuals aged 15–60 years after a rising epidemic in April 1987, and the 485 individuals represented inhabitants of Aasiaat, Maniitsoq, Qaqortoq, and Narsaq. From 330 of these, a blood sample was stored and available for measurement of 25(OH)D3; these samples allowed assessment of time trends in 25(OH)D3 concentrations.

### Collection of blood samples and potential confounders

**Figure 1:**
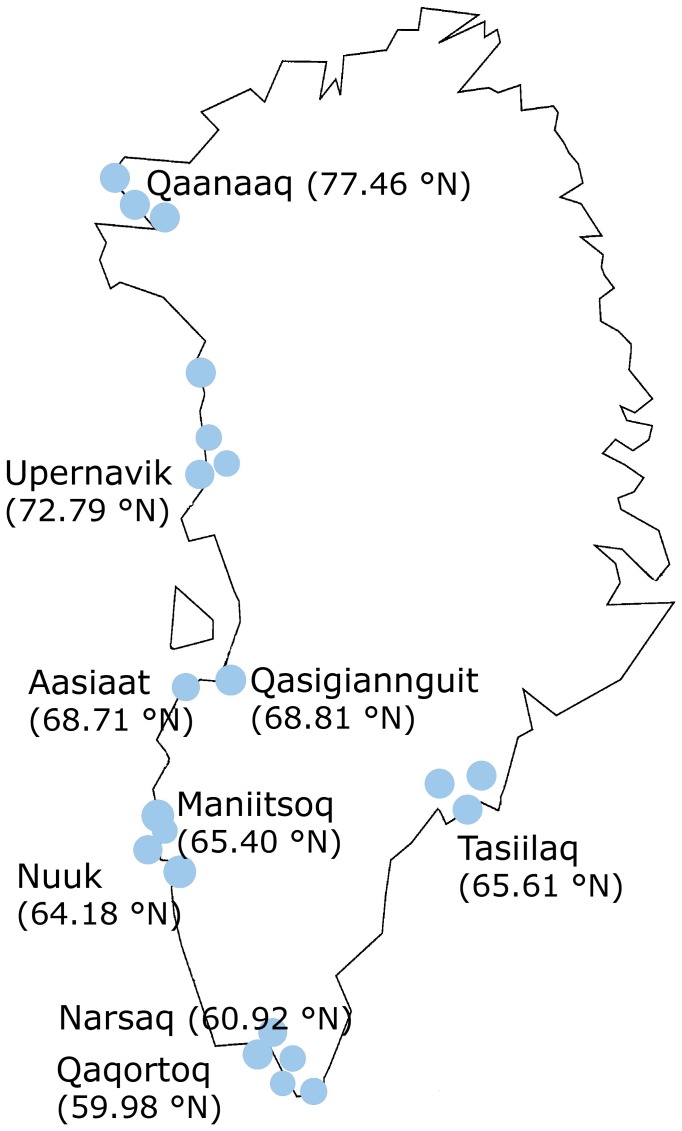
Map of Greenland. The map shows the 9 towns and appurtenant villages where the study participants were living.

Blood samples from 1987 were drawn during May and June. In the IHIT study, blood samples and data on potential confounders were collected by expeditions along the north-west, south and the east coasts of Greenland (**Figure 1**). Samples were collected during all months of the year, except for July, November and December (due to annual leave and inaccessibility) in the period April 2005 to October 2010. Samples were collected in nine towns and 13 villages.

Information on body mass index (BMI) was generated from data on height and weight (weight in kg/(height in m)^2^) obtained from anthropometric measurements, and information on alcohol intake was collected from a self-administered questionnaire. Data on ethnicity, smoking, residence in a town or a village, socioeconomic position, use of vitamin supplements and intake of traditional food were obtained by interview guided questionnaires. Ethnicity (fully or partly Inuit) was based on the grandparent's ethnicity. A participant reporting to have four Inuit grandparents was defined as fully Inuit, whereas a participant reporting to have 1–3 grandparents of non-Inuit descent was defined as partly Inuit. A semi-quantitative food frequency questionnaire including 25 traditional and 43 imported energy-contributing items was used to quantify the food intake. Reported frequency of intake and estimated portion sizes were used when calculating intake in grams/day (g/d). For traditional food, seasonal variation was included in the calculations. Details on collection of dietary data have been described elsewhere [22]. Socioeconomic position was categorized based on educational level and occupation. Information on potential confounders beyond age, gender and latitude was not available from the 1987 sample.

### Vitamin D measurements

Blood samples from the IHIT study were drawn by venipuncture after fasting overnight. Whole blood was allowed to clot and serum was separated by centrifugation for 10 minutes at 3000 rpm. Samples were stored at −20°C until transfer to biobank (Steno Diabetes Center, Gentofte, Denmark). Here, the samples were stored at −80°C until analysis. Blood samples from 1987 were drawn using the same method. These samples were transferred to Statens Serum Institut (Copenhagen, Denmark) where they were tested for syphilis and the remaining serum was stored at −80°C until analysis. IHIT samples were stored for 3–7 years and the 1987-samples were stored for 26 years before thawed and analyzed for 25(OH)D3 and 25(OH)D2. Analyses were performed by liquid chromatography-tandem mass spectrometry (LC-MSMS) using the “MSMS vitamin D” kit from Perkin Elmer (Waltham, MA) as described previously [23]. The method measured both serum 25(OH)D3 and 25(OH)D2, but since concentrations of 25(OH)D2 were negligible, only 25(OH)D3 was considered in the analyses.

### Ethics

At inclusion, IHIT-participants gave their informed written consent to participate in the health investigation. The present study, including use of the stored samples from 1987, was reviewed and approved by the Ethical Review Committee for Greenland. The samples from 1987 stem from a population-based serological syphilis survey [21] and were stored at Statens Serum Institut (Copenhagen, Denmark) and The Danish National Biobank (http://www.ssi.dk/English/Service/AboutSSI/Organization/Organisationchart/Department.aspx?id=e4091b0d-0269-4445-9fe5-9db500a0483e) from where they were procured. Since all study participants gave their informed written consent to participate in the IHIT-study in 2005–2010, the Ethical Review Committee for Greenland waived the need for consent regarding the samples previously collected from the same individuals in 1987.

### Statistical methods

The statistical analyses were performed in STATA 12. The Students' t-test, one-way ANOVA, univariate ANOVA and the χ^2^-test were used to test for differences in geometric means and proportions, as appropriate, and the paired samples t-test was used to test for differences between baseline and follow-up concentrations. For descriptive analysis, serum 25(OH)D3 concentrations were categorized as less than 25 nmol/L, 25–50 nmol/L and 50 nmol/L or more. Ln-transformation of 25(OH)D3 concentrations were used to achieve normal distributions before entered in linear regression models. Beta-coefficients from linear regression models were back-transformed and presented as percentage change in serum 25(OH)D3 per defined unit change in continuous explanatory variables, and percentage difference in geometric mean 25(OH)D3 between a variable category and the reference group for categorical explanatory variables. Quantitative variables were handled as continuous variables. Potential confounders were chosen *a priori* based on current literature and biological plausibility and entered group-wise in four linear regression models. Model 1 was adjusted for age, gender, residence, ethnicity, smoking, alcohol, socioeconomic position, BMI and vitamin supplementation, and the consecutive models were additionally adjusted for intake of traditional diet (model 2), season (model 3) and latitude (model 4). Full case analyses were used. Test for interaction between season and latitude was performed. Only Greenlanders reporting a realistic energy intake in relation to estimates of resting metabolic rate were included in analyses of diet. Thus, based on cut-offs previously defined [24], males who reported an energy intake lower than 3350 kJ/d or higher than 17000 kJ/d, and females reporting an energy intake lower than 2100 kJ/d or higher than 15000 kJ/d were excluded in the linear regression models.

## Results

Of the 2877 participants in the IHIT study and the 330 individuals in the 1987-sample with a serum 25(OH)D3 measurement, 1764 and 306 had complete data on all potential predictors. The mean age of the 1987-sample and the IHIT-sample (2005–2010) was 31.9 years (range 14–62 years) and 44.3 years (range 18–95 years), respectively. The geometric mean serum 25(OH)D3 was 55.8 nmol/L (95% confidence interval [CI]: 53.8; 57.9) in the 1987-sample and 43.4 nmol/L (95% CI: 41.8; 45.7) in the IHIT-sample. In the 1987-sample females tended to have lower serum 25(OH)D3 than males (52.8 nmol/L and 60.5 nmol/L, respectively, p = 0.050, t-test), whereas no gender difference was observed in the IHIT-sample (44.5 and 43.2 nmol/L, respectively, p = 0.212, t-test). Serum 25(OH)D3 increased with age in males and females in both periods and was higher in all categories of age and gender in 1987 compared to 2005–2010. **Table 1** gives the age and gender-specific geometric mean serum 25(OH)D3 concentrations in the two periods 1987 and 2005–2010 and shows the percentage-wise decrease in serum 25(OH)D3 from 1987 to 2005–2010 in the season-matched samples.

**Table 1:** Serum 25(OH)D3 (nmol/L) among 306 individuals examined in May-June 1987 and 745 individuals examined in May-June 2005–2010 by gender and age groups^a^.

		1987			2005-2010		1987 to 2005–2010
	n	Serum 25(OH)D3 (nmol/L)	*P-*value^b^	n	Serum 25(OH)D3 (nmol/L)	*P-*value^b^	Decrease
Males							
Age (years)			<0.001			<0.001	
18–29	50	47.8 (46.6; 49.0)		44	29.1 (27.9; 30.3)		39%
30–49	65	72.9 (71.8; 74.0)		124	34.4 (33.3; 35.5)		53%
50–69	10	116.8 (115.4; 118.2)		96	49.5 (48.4; 50.6)		58%
70+	0	-		24	49.3 (48.1; 50.5)		-
Females							
Age (years)			<0.001			<0.001	
18–29	84	44.2 (43.0; 45.3)		76	29.4 (28.3; 30.5)		33%
30–49	83	68.5 (67.4; 69.6)		238	32.6 (31.5; 33.7)		52%
50–69	14	73.4 (72.3; 74.5)		110	50.0 (48.9; 51.1)		32%
70+	0	-		33	48.6 (47.4; 49.8)		-

a Data are geometric mean (95% confidence interval) adjusted for latitude.

b *P* values were calculated by using univariate ANOVA for measure of differences between groups.

**Figure 2:**
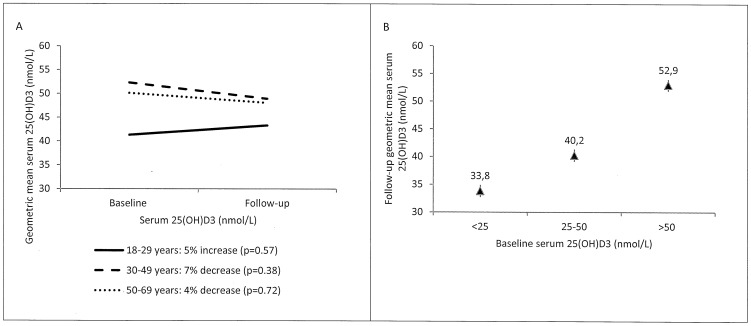
Baseline and follow-up concentrations of vitamin D. (A) Geometric mean serum 25(OH)D3 concentration among 138 individuals with a baseline (1987) and a follow-up (2005–2010) measurement in May-June 1987 and in May-June 2005–2010, respectively, by age group, and (B) geometric mean serum follow-up 25(OH)D3 concentration with 95% confidence interval by baseline concentration (deficiency: <25 nmol/L; insufficiency: 25–50; sufficiency:>50 nmol/L) after adjustment for age, gender, latitude and month of follow-up sampling among 309 individuals.

We also evaluated the development in serum 25(OH)D3 concentration within the cohort of individuals with two measurements of serum 25(OH)D3, and the association between baseline 25(OH)D3 and the follow-up concentration. **Figure 2A** gives the baseline and follow-up 25(OH)D3 concentrations and shows a small and insignificant change from 1987 to 2005–2010 observed within the cohort of participants with season-matched baseline and follow-up measurements. The geometric mean serum 25(OH)D3 concentration at follow-up was positively associated with the baseline serum 25(OH)D3 concentrations (p<0.001). The geometric mean concentrations at follow-up after adjustment for age, gender, latitude and month of follow-up sampling are shown in **Figure 2B**.

Among the IHIT participants, the overall geometric mean serum 25(OH)D3 was lowest (30.7 nmol/L; CI: 29.7; 31.7) in the youngest age group (18–29 years), and, correspondingly, the prevalence of concentrations of 25–50 nmol/L (39%) and less than 25 nmol/L (37%) was highest in this group, whereas concentrations above 50 nmol/L were most prevalent at ages 50 years or more. **Table 2** gives the age and gender specific geometric mean serum 25(OH)D3 concentrations and the prevalence of concentrations above 50 nmol/L, 25–50 nmol/L and less than 25 nmol/L. A similar pattern was observed in the sample from 1987, although the prevalence of concentrations less than 25 nmol/L was approximately one third of what was found in the IHIT sample (14% for males and 12% for females) in the youngest age-group, and concentrations above 50 nmol/L were measured in 100% of the males and 86% of the females aged 50 years or more (data shown in Table S1).

**Table 2:** Serum 25(OH)D3 (nmol/L) and prevalence of concentrations above 50 nmol/L, 25–50 nmol/L and less than 25 nmol/L among 2877 individuals included in the IHIT-study, 2005–2010, by gender and age groups.

	n	Serum 25(OH)D3 (nmol/L)^a^	*P*-value^c^	>50 nmol/L^b^	25–50 nmol/L^b^	<25 nmol/L^b^	*P*-value^c^
Males							
Age (years)			<0.001				<0.001
18–29	221	28.5 (27.4; 29.6)		23	34	43	
30–49	572	42.2 (41.2; 43.3)		41	40	19	
50–69	391	58.3 (57.2; 59.4)		68	27	6	
70+	72	59.7 (58.6; 60.8)		72	22	6	
Females							
Age (years)			<0.001				<0.001
18–29	314	32.3 (31.2; 33.4)		25	42	33	
30–49	791	39.4 (38.4; 40.4)		35	44	21	
50–69	422	59.7 (58.7; 60.8)		70	26	4	
70+	94	57.7 (56.6; 58.8)		66	27	7	

a Data are geometric mean (95% confidence interval).

b % of participants with serum 25(OH)D3 concentration within cutoff.

c *P* values were calculated by using univariate ANOVA and chi-square test for measure of differences between groups.

**Figure 3:**
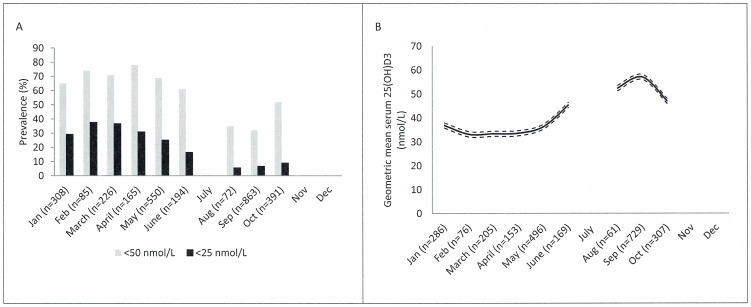
Seasonal variation in vitamin D. (A) Seasonal variation in vitamin D insufficiency (<50 nmol/L) and deficiency (<25 nmol/L) measured as serum 25(OH)D3 among 2854 adults included in the IHIT study, 2005–2010, and (B) geometric mean serum 25(OH)D3 concentration (nmol/L) with 95% confidence interval by month after adjustment for age, gender and intake of traditional food among 2482 adults included in the IHIT study, 2005–2010.

A characteristic seasonal variation in the prevalence of 25(OH)D3 concentrations less than 50 nmol/L and less than 25 nmol/L was observed (**Figure 3A**). The highest prevalences of concentrations less than 50 nmol/L and 25 nmol/L appeared in February to April and the lowest in August to September. There was a similar seasonal variation in serum 25(OH)D3 after adjustment for age, gender, and intake of traditional food (**Figure 3B**). Geometric mean serum 25(OH)D3 concentrations varied significantly (range 33.2–57.1 nmol/L, p<0.001) over the nine months, with the highest concentrations in the summer and autumn.

As presented in **Table 3**, autumn season was a strong predictor of high serum 25(OH)D3 concentrations (47.1%; 95% CI: 35.8; 59.2; winter was reference), whereas increasing latitude predicted decreasing concentrations (−0.7%; 95% CI: −1.3; −0.2). There was no interaction between season and latitude. Age (2.0%; 95% CI: 1.7; 2.2), female gender (7.1%; 95% CI: 2.0; 12.5), smoking (−11.6%; 95% CI: −16.2; −6.9), alcohol (0.2%; 95% CI: 0.0; 0.4), BMI (−0.6; 95% CI: −1.1; −0.2), supplementation with vitamin D or multivitamins (12.0%; 95% CI: 4.2; 20.4), and intake of traditional food (10.0%; 95% CI: 7.9; 12.1) were associated with serum 25(OH)D3 after full adjustment. Residence in a village versus a town appeared to be associated with higher serum 25(OH)D3, but the association was attenuated and became insignificant after adjustment for traditional food, season and latitude. Likewise, the association between socioeconomic position and serum 25(OH)D3 disappeared after adjustment for traditional food, season and latitude. In general, adjustment for latitude only had small effects on the estimates.

**Table 3:** Predictors of serum 25(OH)D3 (nmol/L) expressed as percentage change per defined unit change in the explanatory variable^a^.

	Model 1		Model 2		Model 3		Model 4	
	Adjusted for age, gender, residence, ethnicity, smoking, alcohol, socioeconomic position, BMI and supplementation		Additionally adjusted for traditional food		Additionally adjusted for season		Additionally adjusted for latitude	
	(n = 1817)		(n = 1779)		(n = 1764)		(n = 1764)	
	% (95% CI)	*P* value	% (95% CI)	*P* value	% (95% CI)	*P* value	% (95% CI)	*P* value
Age (per year)	2.7 (1.9; 2.3)	<0.001	1.9 (1.7; 2.1)	<0.001	2.0 (1.7; 2.2)	<0.001	2.0 (1.7; 2.2)	<0.001
Gender		0.100		0.004		0.010		0.006
Male	ref		ref		ref		ref	
Female	4.4 (−0.8; 10.0)		7.8 (2.4; 13.4)		6.6 (1.5; 12.0)		7.1 (2.0; 12.5)	
Residence		<0.001		<0.001		1.110		0.161
Town	ref		ref		ref		ref	
Village	50.9 (41.7; 60.8)		35.3 (26.8; 44.3)		6.1 (−1.3; 14.2)		5.4 (−2.7; 13.3)	
Ethnicity^b^		0.316		0.138		0.289		0.386
Partly Inuit	ref		ref		ref		ref	
Fully Inuit	−3.4 (−9.8; 3.4)		−5.0 (−11.2; 1.7)		−3.4 (−9.5; 3.0)		−2.8 (−9.0; 3.7)	
Smoking		<0.001		<0.001		<0.001		<0.001
No	ref		ref		ref		ref	
Yes	−11.0 (−15.5; −5.5)		−10.5 (−15.3; −5.4)		−12.0 (−16.2; −6.9)		−11.6 (−16.2; −6.9)	
Alcohol (per drinks/week)	0.3 (0.1; 0.5)	0.001	0.3 (0.1; 0.5)	0.004	0.2 (0.0; 0.4)	0.022	0.2 (0.0; 0.4)	0.038
Socioeconomic position		0.008		0.220		0.056		0.057
High^c^	ref		ref		ref		ref	
Skilled	−1.0 (−9.0; 7.6)		−0.7 (−8.5; 7.8)		−2.6 (−10.0; 5.4)		−2.2 (−9.6; 5.9)	
Unskilled	1.39 (−6.5; 9.9)		0.5 (−7.2; 7.8)		0.5 (−7.0; 8.5)		1.6 (−5.9; 9.8)	
Hunters/fishermen	16.9 (3.7; 32.0)		8.0 (−4.1; 21.7)		11.2 (−0.9; 24.7)		12.3 (0.1; 9; 25.9)	
Unemploid^d^	9.3 (−0.3; 19.0)		6.7 (−1.9; 16.1)		6.3 (−2.5; 15.3)		6.7 (−1.6; 15.7)	
BMI (per kg/m^2^)	−0.6 (−1.1; −0.1)	0.019	−0.7 (−1.2; −0.2)	0.009	−0.7 (−1.1; −0.2)	0.008	−0.6 (−1.1; −0.2)	0.009
Supplementation^e^		0.033		0.017		0.002		0.002
No	ref		ref		ref		ref	
yes	8.7 (0.7; 17.3)		9.6 (1.6; 18.2)		12.3 (4.5; 20.7)		12.0 (4.2; 20.4)	
Traditional food intake (per 100 g/d)^f^	-	-	11.6 (9.5; 13.8)	<0.001	9.6 (7.6; 11.6)	<0.001	10.0 (7.9; 12.1)	<0.001
Season^g^		-		-		<0.001		<0.001
Winter	-		-		ref		ref	
Spring	-		-		−3.6 (−10.2; 3.5)		−3.9 (−10.5; 3.1)	
Summer	-		-		28.7 (17.0; 41.5)		31.0 (19.1; 44.2)	
Autumn	-		-		45.2 (34.2; 57.1)		47.1 (35.8; 59.2)	
Latitude (per degree)	-	-	-	-	-	-	−0.7 (−1.3; −0.2)	0.008

a Only Inuit in the IHIT-study reporting a realistic energy intake were included in the analyses (n = 2570).

b A participant was perceived as fully Inuit if he/she reported him/her-self to be Inuit and to have four Inuit grandparents, and as partly Inuit if 1–3 of the grandparents were not Inuit.

c Work that presupposes a medium-long education (white collar employees).

d Students, individuals receiving transfer payment, unemployed and stay-at-home individuals.

e Vitamin D or multivitamins.

f Traditional food was defined as seal, blubber (boiled or frozen), white whale, other whales, walrus, mattak (whale skin), offal from seal, cod, halibut, capelin, trout, dried fish and other fish.

g Winter: January + February; Spring: March-May; Summer: June + August; Autumn: September + October. Blood samples (and thereby vitamin D measurements) were not available from July, November and December due to summer annual leave and rough weather conditions during winter.

## Discussion

Our study showed a marked decline in serum vitamin D status among Inuit in Greenland from 1987 to 2005–2010 in all age groups. The prevalence of serum 25(OH)D3 concentrations of 50 nmol/L or less was highest in the youngest age groups in 2005–2010, and considerably higher than in 1987. This finding could very well be a consequence of the fact that intake of the traditional vitamin D rich diet to a large extent has been substituted by imported foods [25], and it could be speculated that increased time spent on sedentary activities [3] is correlated with a decrease in time spent on outdoor activities. Intake of traditional food in Greenland is highest among the oldest age groups (data shown in Table S2) and the increase in serum 25(OH)D3 with age could suggest a significant dietary contribution to the vitamin D status compensating for the age-dependent impaired capability of dermal production, which in western populations results in decreasing vitamin D with increasing age [26], [27]. This is supported by the almost unchanged 25(OH)D3 concentrations from baseline (1987) to follow-up (2005–2010) observed among the cohort participants with two season-matched measurements, where at least in the oldest age group, a more profound decline would be expected over the 18–23 years. However, age was positively associated with 25(OH)D3 status even after adjusting for traditional food intake, indicating that other factors, possibly time spent outdoors, may play a crucial role. Accordingly, higher 25(OH)D3 concentration among females may be associated with intentional higher sun exposure as compared with males.

Our study could not show a cohort effect on 25(OH)D3 status but a strong period effect was observed. This may indicate that lifestyle changes mainly have affected the younger generations, whereas traditional dietary patterns have been maintained within the older generations.

We found that serum 25(OH)D3 varied with season as reflected in a significant increase in 25(OH)D3 concentrations and lower prevalence of concentrations less than 50 nmol/L and less than 25 nmol/L during summer and autumn. This indicates that dermal production of vitamin D occurs in Greenland, contributing to the vitamin D status during summer and autumn, despite the high solar zenith angle. There was no interaction between season and latitude, thus, the observed seasonality appears to apply at all latitudes. Increasing latitude was independently associated with lower serum 25(OH)D3, suggesting decreasing vitamin D concentrations in the seasonal pattern, with increasing latitude. Thus, the reduced dermal production of vitamin D at increasing latitudes appears not to be compensated for by dietary intake.

The finding of an influence by season, indicating dermal production, on the 25(OH)D3 status in the Arctic is in accordance with the conclusions drawn in a previous study in Greenland [14], and the seasonality pattern is similar to that observed in western populations [28], [29]. It is, however, important to notice that intake of vitamin D through the diet also increases during summer and autumn in our study population (data shown in Figure S1), and despite adjustment for vitamin D intake through traditional food, an influence of residual confounding or potential other dietary sources on the observed seasonality cannot be excluded.

As expected, traditional diet and vitamin supplementation were strongly associated with 25(OH)D3 concentration, which confirms the significant role of vitamin D intake on the vitamin D status in the Arctic. Likewise, our finding of lower 25(OH)D3 concentrations among smokers than non-smokers was expected and in accordance with the assumption that smoking affects the vitamin D metabolism through impairment of 25-hydroxylase activity in the liver [30], [31]. Surprisingly, we found a positive association between alcohol consumption and 25(OH)D3 status. This finding is, however, supported by similar observations from a large Norwegian health study [32]. Although speculative, this association could possibly be explained by alcohol induced impairment of the secretion of parathyroid hormone [32], [33], which increases the activity of 25(OH)D3 1α-hydroxylase, the enzyme responsible for the conversion of 25(OH)D3 to 1,25(OH)2D3 [34]. Impaired conversion of 25(OH)D3 to 1,25(OH)2D3 might result in increased 25(OH)D3 concentrations (which were measured in this study), and probably reduced concentrations of 1,25(OH)2D3 (not measured), with increasing alcohol intake.

A negative association between vitamin D status and BMI is well known [35], [36] and often reported as lower concentrations of 25(OH)D among individuals with obesity [37], [38] or high body fat percentage [39]. Accordingly, we observed decreasing serum 25(OH)D3 with increasing BMI. This negative association is likely due to the depositing of vitamin D in fat tissue, which is supported by reported increments in serum 25(OH)D after weight loss as a result of surgical intervention [36], [40]–[42].

Overall, socioeconomic position appeared not to be associated with 25(OH)D3 status after adjustment for intake of traditional diet, season and latitude, although hunters/fishermen had a remarkably higher vitamin D status than white collar employees (reference group). A combination of a possible higher sun exposure among hunters/fishermen and potential residual confounding effects from traditional diet could possibly account for this difference.

Serum 25(OH)D3 in 1987 and in 2005–2010 was measured in blood samples exposed to long storage, which introduces the potential risk of decay. However, the content of 25(OH)D3 appears to be stable in serum despite exaggerated conditions [43] and long storage periods [44], and higher contents in 1987-samples disfavor this hypothesis. Thus, although stored for 3–26 years, the concentrations reported in samples from 1987 and 2005–2010 are likely indicative of the concentrations at the time of sampling, and in the unexpected case of decay, the observed decrease in serum 25(OH)D3 over time would be underestimated.

Strengths of the study included assessment of vitamin D status in a large stratified random sample of Inuit in Greenland across various latitudes. Furthermore, we had the opportunity to estimate the time trend in vitamin D status in the period 1987 to 2005–2010, and to assess the association between baseline serum concentrations of 25(OH)D3 and follow-up concentrations, by using blood samples collected from a sub-sample of the IHIT study population in 1987. Blood samples were collected across all seasons and information on sociodemographic and lifestyle factors were available from questionnaires, which enabled us to control for potential confounding. The study also had potential limitations. Determination of serum 25(OH)D3 status based on one measurement in the IHIT study may have introduced measurement error, and the fact that 25(OH)D3 measurements were not available from July, November and December may have resulted in over- and under-estimation of serum 25(OH)D3 and the prevalence of concentrations less than 50 nmol/L and 25 nmol/L in summer and winter seasons. In general, the relevance of cut-points for 25(OH)D3 concentrations used in this and various other studies, is subject to discussion; we decided to use cut-points defined by the Danish National Board of Health as sufficiency (>50 nmol/L), insufficiency (25–50 nmol/L) and deficiency (<25 nmol/L) in some of the analyses of our Inuit population since the frequency distribution of serum 25(OH)D3 concentrations did not differ much from those presented for other Nordic populations [28], [32]. Due to the observational nature of the study, causality of associations cannot be determined, and we cannot exclude the possibility that our results are affected by confounders not adjusted for. For instance, we did not have data on time spent outdoors.

Although generalizability of our findings to populations at lower latitudes may be uncertain, it is likely that other indigenous populations living at high latitudes and undergoing profound lifestyle changes may be subjects to equivalent changes in vitamin D status, and predictors of low vitamin D status may be similar.

We conclude that the vitamin D status of the general adult Inuit population in Greenland is low, especially among persons below the age of 30. The remarkable decrease in serum 25(OH)D3 from 1987 to 2005–2010 is likely to indicate that traditional diet was formerly responsible for sustaining a healthy vitamin D status among Inuit. Increased consumption of fatty fish, which is already recommended by the Nutritional Board of Greenland, together with increased cautious sun exposure would probably improve the current situation and vitamin D supplementation might be considered for vulnerable population groups.

## Supporting Information

Figure S1 **Seasonal variation in traditional food intake.** Geometric mean intake of traditional food (g/d) with 95% confidence interval by month after adjustment for age, gender and latitude among 2643 adults included in the IHIT study, 2005–2010.
(TIF)

Table S1 **Prevalence of serum 25(OH)D3 concentrations above 50 nmol/L, 25–50 nmol/L, and less than 25 nmol/L among 306 individuals examined in 1987, by gender and age groups.**
(DOCX)

Table S2 **Geometric mean intake of traditional food (g/d) among 2683 individuals included in the IHIT-study, 2005–2010, by gender and age groups.**
(DOCX)

## References

[1] Young TK, Bjerregaard P (2008) Health Transitions in Arctic Populations, Edited by T. Kue and Peter Bjerregaard, University of Toronto Press Incorporated.

[2] Bjerregaard P, Young TK, Dewailly E, Ebbesson SO (2004) Indigenous health in the Arctic: an overview of the circumpolar Inuit population. Scand J Public Health 32:390–395. 15513673 10.1080/14034940410028398

[3] Dahl-Pedersen IK, Joergensen ME, Bjerregaard P (2011) Physical activity patterns in Greenland: a country in transition. Scand J Public Health 39:678–686. 21948977 10.1177/1403494811420486

[4] Jørgensen ME, Bjerregaard P, Borch-Johnsen K, Backer V, Becker U, et al (2002) Diabetes and impaired glucose tolerance among the Inuit population of Greenland. Diabetes Care 25:1766–1771. 12351475 10.2337/diacare.25.10.1766

[5] Jørgensen ME, Glümer C, Bjerregaard P, Gyntelberg F, Jørgensen T, et al (2003) Obesity and central fat pattern among Greenland Inuit and a general population of Denmark (Inter99): Relationship to metabolic risk factors. Int J Obes 27:1507–1515. 10.1038/sj.ijo.0802434 14634682

[6] Jørgensen ME, Bjerregaard P, Kjærsgaard JJ, Borch-Johnsen K (2008) High prevalence of markers of coronary heart disease among Greenland Inuit. Atherosclerosis 196:772–778. 17306273 10.1016/j.atherosclerosis.2007.01.008

[7] Holick MF (2007) Vitamin D deficiency. N Engl J Med 357:266–281. 17634462 10.1056/NEJMra070553

[8] Bell DS (2011) Protean manifestations of vitamin D deficiency, part 2: deficiency and its association with autoimmune disease, cancer, infection, asthma, dermopathies, insulin resistance, and type 2 diabetes. South Med J 104:335–339. 21606712 10.1097/01.SMJ.0000397893.94525.0e

[9] Jahnsen J, Falch JA, Mowinckle ZP, Aadland E (2002) Vitamin D status, parathyroid hormone and bone mineral density in patients with inflammatory bowel disease. Scand J Gastroenterol 37:192–197. 11843057 10.1080/003655202753416876

[10] Matilla C, Knekt P, Männistö, Rissanen H (2007) Serum 25-hydroxyvitamin D concentration and subsequent risk of type 2 diabetes. Diabetes Care 30:2569–2570. 17626891 10.2337/dc07-0292

[11] Nielsen NO, Skifte T, Andersson M, Wohlfahrt J, Søborg B, et al (2010) Both high and low serum vitamin D concentrations are associated with tuberculosis: a case-control study in Greenland. Br J Nutr 104:1487–1491. 20553638 10.1017/S0007114510002333

[12] Forouhi NG, Luan J, Cooper A, Boucher BJ, Wareham NJ (2008) Baseline serum 25-hydroxy vitamin D is predictive of future glycemic status and insulin resistance. The Medical Research Council ELY Prospective Study 1990–2000. Diabetes 57:2619–2625. 18591391 10.2337/db08-0593 PMC2551670

[13] Webb AR (2006) Who, what, where and when – influences on cutaneous vitamin D synthesis. Prog Biophys Mol Biol 92:17–25. 16766240 10.1016/j.pbiomolbio.2006.02.004

[14] Andersen S, Jacobsen A, Laurberg P (2012) Vitamin D status in North Greenland is influenced by diet and season: indicators of dermal 25-hydroxy vitamin D production north of the Arctic Circle. Br J Nutr 110:50–57 10.1017/S0007114512004709 23182389

[15] Kuhlein HV, Receveur O, Soueida R, Berti PR (2007) Unique patterns of dietary adequacy in three cultures of Canadian Arctic indigenous peoples. Publ Health Nutr 11:349–360. 10.1017/S1368980007000353 17610753

[16] Andersen S, Hvingel B, Kleinschmidt K, Jørgensen T, Laurberg P (2005) Changes in iodine excretion in 50–69-y-old denizens of an Arctic society in transition and iodine excretion as a biomarker of the frequency of consumption of traditional Inuit foods. Am J Clin Nutr 81:656–663. 15755836 10.1093/ajcn/81.3.656

[17] Rejnmark L, Jørgensen ME, Pedersen MB, Hansen JC, Heickendorff L, et al (2004) Vitamin D insufficiency in Greenlanders on a westernized fare: Ethnic differences in calcitropic hormones between Greenlanders and Danes. Calcif Tissue Int 74:255–263. 14708040 10.1007/s00223-003-0110-9

[18] Andersen S, Laurberg P, Hvingel B, Kleinschmidt K, Heickendorff L, et al (2013) Vitamin D status in Greenland is influenced by diet and ethnicity: a population-based survey in an Arctic society in transition. Br J Clin Nutr 109:928–935. 10.1017/S0007114512002097 22682501

[19] Dahl-Petersen IK, Bjerregaard P, Brage S, Jørgensen ME (2013) Physical activity energy expenditure is associated with 2-h insulin independently of obesity among Inuit in Greenland. Diabetes Res Clin Pract 102:242–249. 24176243 10.1016/j.diabres.2013.10.004

[20] Bjerregaard P (2010) Inuit Health in Transition – Greenland survey 2005-2010. Population sample and survey methods. SIF Writings on Greenland vol. 19 . Internet: http://www.si-folkesundhed.dk/upload/inuit_health_in_transition_greenland_methods_5_2nd_revision.pdf. Accessed 04 July 2014.

[21] Misfeldt J, Jørgensen BB, Larsen SO (1988) A serological mass examination for syphilis in Greenland in 1987. Atc Med Res 47:173–178. 3214507

[22] Jeppesen C, Bjerregaard P, Jørgensen ME (2012) Dietary patterns in Greenland and their relationship with type 2 diabetes mellitus and glucose intolerance. Public Health Nutr 17:462–470 10.1017/S136898001300013X PMC10282280 23399043

[23] Nielsen NO, Strøm M, Boyd HA, Andersen EW, Wohlfahrt J, et al (2013) Vitamin D status during pregnancy and the risk of subsequent postpartum depression: A case-control study. PlosOne 8:e80686 10.1371/journal.pone.0080686 PMC3842313 24312237

[24] Willet W (2013) Issues in analysis and presentation of dietary data. In: Hofman A, Marmot M, Samet J, Savitz DZeditors. Nutritional Epidemiology 3 ^rd^ edOxford University Press, pp. 306.

[25] Deutch B, Dyerberg J, Pedersen HS, Aschlund E, Hansen JC (2007) Traditional and modern Greenlandic food – dietary composition, nutrients and contaminants. Sci Total Environ 384:106–119. 17629548 10.1016/j.scitotenv.2007.05.042

[26] Holick MF (2004) Sunlight and vitamin D for bone health and prevention of autoimmune diseases, cancers, and cardiovascular disease. Am J Clin Nutr 80 (suppl): 1678S–1688S. 10.1093/ajcn/80.6.1678S 15585788

[27] Ginde AA, Liu MC, Camargo CA (2009) Demographic differences and trends of vitamin D insufficiency in the US population, 1998–2004. Arch Intern Med 169:626–632. 19307527 10.1001/archinternmed.2008.604 PMC3447083

[28] Thuesen B, Husemoen L, Fenger M, Jakobsen J, Schwarz P, et al (2012) Determination of vitamin D status in a general population of Danish adults. Bone 50:605–610 10.1016/j.bone.2011.12.016 22227435

[29] Cannell JJ, Hollis BW, Sorensen MB, Taft TN, Anderson JJB (2009) Athletic performance and vitamin D. Med Sci Sports Exerc 41:1102–1110. 19346976 10.1249/MSS.0b013e3181930c2b

[30] Abate M, Vanni D, Pantalone A, Salini V (2013) Cigarette smoking and musculoskeletal disorders. Muscles Ligaments Tendons J 3:63–69 10.11138/mltj/2013.3.2.063 23888288 PMC3711704

[31] Need AG, Kemp A, Giles N, Morris HA, Horowitz M, et al (2002) Relationships between intestinal calcium absorption, serum vitamin D metabolites and smoking in postmenopausal women. Osteoporos Int 13:83–88. 11883410 10.1007/s198-002-8342-9

[32] Larose TC, Chen Y, Camargo CA, Langhammer A, Romundstad P, et al (2014) Factors associated with vitamin D deficiency in a Norwegian population: the HUNT study. J Epidemiol Community Health 68:165–170 10.1136/jech-2013-202587 24197920

[33] McCarty MF, Thomas CA (2003) PTH excess may promote weight gain by impeding catecholamine-induced lipolysis-implications for the impact of calcium, vitamin D, and alcohol on body weight. Med Hypotheses 61:535–542. 14592784 10.1016/s0306-9877(03)00227-5

[34] Eyles D, Smith S, Kinobe R, Hewison M, McGrath JJ (2005) Distribution of the vitamin D receptor and 1 alpha-hydroxylase in human brain. J Chem Neu 29:21–30. 10.1016/j.jchemneu.2004.08.006 15589699

[35] Jorde R, Sneve M, Emaus N, Figenschau Y, Grimnes G (2010) Cross-sectional and longitudinal relation between serum 25-hydroxyvitamin D and body mass index: the Tromsø study. Eur J Nutr 49:401–407. 20204652 10.1007/s00394-010-0098-7

[36] Friis H, Range N, Changalucha J, PrayGod G, Jeremiah K, et al (2013) Vitamin D status among pulmonary TB patients and non-TB controls: A cross-sectional study from Mwanza, Tanzania. PlosOne 8: e81142 10.1371/journal.pone.0081142 PMC3855700 24324666

[37] Wortsman J, Matsuoka LY, Chen TC, Lu Z, Holick MF (2000) Decreased bioavailability of vitamin D in obesity. Am J Clin Nutr 72:690–693. 10966885 10.1093/ajcn/72.3.690

[38] Jamal-Allial A, Griffith JL, Tucker KL (2013) The longitudinal association of vitamin D serum concentrations & adiposity phenotype. J Steroid Biochem Mol Biol [Epub ahead of print]. doi:10.1016/j.jsbmb.2013.12.004. 10.1016/j.jsbmb.2013.12.004 PMC4055547 24333795

[39] Arunabh S, Pollack S, Yeh J, Aloia JF (2003) Body fat content and 25-hydroxyvitamin D concentrations in healthy women. J Clin Endocrinol Metab 88:157–161. 12519845 10.1210/jc.2002-020978

[40] Sulistyoningrum DC, Green TJ, Lear SA, Devlin AM (2012) Ethnic-specific differences in vitamin D status is associated with adiposity. PlosOne 7:e43159. 10.1371/journal.pone.0043159 PMC3430647 22952641

[41] Durup D, Jørgensen HL, Christensen J, Schwarz P, Heegaard AM (2012) A reverse J-shaped association of all-cause mortality with serum 25-hydroxyvitamin D in general practice, the CopD study. J Clin Endocrin Metab 97:2644–2652. 10.1210/jc.2012-1176 22573406

[42] Kouda K, Nakamura H, Fujita Y, Ohara K, Iki M (2013) Vitamin D status and body fat measured by dual-energy X-ray absorptiometry in a general population of Japanese children. Nutrition 29:1204–1208. 23800567 10.1016/j.nut.2013.03.010

[43] Lewis JG, Elder PA (2008) Serum 25-OH vitamin D2 and D3 are stable under exaggerated conditions. Clinical Chemistry 54:1931–1932. 18957567 10.1373/clinchem.2008.111526

[44] Agborsangaya C, Toriola AT, Grankvist K, Surcel H-M, Holl K, et al (2010) The effects of storage time and sampling season on the stability of serum 25-hydroxy vitamin D and androstenedione. Nutr Cancer 62:51–57. 20043259 10.1080/01635580903191460

