# Supplementary material for: Decrease in Vitamin D Status in the Greenlandic Adult Population from 1987–2010
Source: PLoS One. 2014 Dec 2;9(12):e112949. doi: 10.1371/journal.pone.0112949 (PMC4252033; doi:10.1371/journal.pone.0112949)
Supplement: Table S1 — Prevalence of serum 25(OH)D3 concentrations above 50 nmol/L, 25–50 nmol/L, and less than 25 nmol/L among 306 individuals examined in 1987, by gender and age groups. (DOCX) [file pone.0112949.s002.docx]

**Table S1.** Prevalence of serum 25(OH)D3 concentrations above 50 nmol/L, 25-50 nmol/L, and less than 25 nmol/L among 306 individuals examined in 1987, by gender and age groups.

|  | n | >50 nmol/L^a^ | 25-50 nmol/L^a^ | <25 nmol/L^a^ | *P*-value^b^ |
| --- | --- | --- | --- | --- | --- |
| Males |  |  |  |  |  |
| Age (years) |  |  |  |  | 0.003 |
| 18-29 | 50 | 48 | 38 | 14 |  |
| 30-49 | 65 | 75 | 22 | 3 |  |
| 50-69 | 10 | 100 | 0 | 0 |  |
| Females |  |  |  |  |  |
| Age (years) |  |  |  |  | 0.005 |
| 18-29 | 84 | 51 | 37 | 12 |  |
| 30-49 | 83 | 74 | 24 | 2 |  |
| 50-69 | 14 | 86 | 7 | 7 |  |

^a^ % of participants with serum 25(OH)D3 concentration within cutoff.

^C^ *P* values were calculated by using chi-square test for measure of differences between groups.
