# Supplementary material for: Decrease in Vitamin D Status in the Greenlandic Adult Population from 1987–2010
Source: PLoS One. 2014 Dec 2;9(12):e112949. doi: 10.1371/journal.pone.0112949 (PMC4252033; doi:10.1371/journal.pone.0112949)
Supplement: Table S2 — Geometric mean intake of traditional food (g/d) among 2683 individuals included in the IHIT-study, 2005–2010, by gender and age groups. (DOCX) [file pone.0112949.s003.docx]

**Table S2.** Geometric mean intake of traditional food (g/d) among 2683 individuals included in the IHIT-study, 2005-2010, by gender and age groups.

|  | n | Intake of traditional food (g/d)^a^ | *P*-value^b^ |
| --- | --- | --- | --- |
| Males |  |  |  |
| Age (years) |  |  | <0.001 |
| 18-29 | 193 | 64.1 (63.0; 65.3) |  |
| 30-49 | 524 | 116.3 (115.2; 117.4) |  |
| 50-69 | 353 | 154.8 (153.7; 155.9) |  |
| 70+ | 71 | 172.4 (171.1; 173.7) |  |
| Females |  |  |  |
| Age (years) |  |  | <0.001 |
| 18-29 | 279 | 58.8 (57.7; 59.9) |  |
| 30-49 | 747 | 78.7 (77.6; 79.8) |  |
| 50-69 | 421 | 98.9 (97.8; 100.0) |  |
| 70+ | 95 | 80.7 (79.5; 81.9) |  |

^a^ Data are geometric mean (95% confidence interval).

^b^ *P* values were calculated by using univariate ANOVA for measure of differences between groups.
